# Artificial intelligence and multi-omics nominate TAZ as an insomnia-related diagnostic and druggable target for Parkinson’s disease patients

**DOI:** 10.3389/fnagi.2026.1727472

**Published:** 2026-02-04

**Authors:** Wenjing Ma

**Affiliations:** Beijing Chaoyang Gaobeidian Community Health Service Center, Beijing, China

**Keywords:** artificial intelligence, insomnia, multi-omics, Parkinson’s disease, predictive model, TAZ

## Abstract

**Background:**

Insomnia is one of the most common non-motor comorbidities of Parkinson’s disease (PD) and often before the onset of motor symptoms. Identifying the molecular mechanisms of insomnia may facilitate the early diagnosis of PD and contribute to therapeutic development.

**Methods:**

Five human PD substantia nigra (SN) bulk-seq datasets (GSE20141, GSE7621, GSE20164, GSE20163, and GSE20333), with an insomnia-related gene list, were acquired from GEO and Genecard databases. First, the integration of GSE20141 and GSE7621 was analyzed to identify insomnia-related DEGs using limma and the WGCNA framework. GSE20164 and GSE20163 combination were used as a training set for insomnia-related hub gene recognition. Furthermore, the aforementioned four datasets, along with an independent validation set (GSE20333), were cross-validated for insomnia-related diagnostic model construction. The human PD-SN single-cell profile (GSE140231) was utilized for exploring the mechanisms underlying the heterogeneity of insomnia-related hub genes in spatial and temporal contexts. Furthermore, a cutting-edge artificial intelligence (AI)-driven framework (DrugRefLector) and molecular docking techniques was used to identify an optimal agent for the treatment of PD based on the GSE20164 and GSE20163 integrated dataset. Finally, an *in vitro* q-RT-PCR experiment was conducted to estimate the targeted gene expression.

**Results:**

TAZ (WWTR1) is associated with the increased expression of insomnia-related diagnostic markers linked to PD pathogenesis, mainly in neurons, and has excellent predictive performance for PD diagnosis. Furthermore, BRD-K97481123 can be considered as a potential therapeutic agent for the treatment of PD by targeting TAZ.

**Conclusion:**

By integrating AI pipelines and multi-omics, our study first traced TAZ mechanisms in PD pathogenesis and elaborated on TAZ’s predictive and druggable potential for PD patients.

## 1 Introduction

Parkinson’s disease (PD) is a common, heterogeneous neurodegenerative disorder with a rising global prevalence (Tolosa et al., 2021). PD is characterized by the progressive degeneration of dopaminergic neurons in the substantia nigra (SN) and the formation of pathological *α*-synuclein aggregates, known as Lewy bodies (Hayes, 2019). The clinical features of PD encompass various motor symptoms, such as tremors and rigidity, and non-motor symptoms, such as olfactory impairment, depressive complications, and insomnia (Kalia and Lang, 2015). The initiation of PD arises from a multifaceted interaction of elements, which include genetic predisposition and environmental factors (Bloem et al., 2021). Although advancements have been made in elucidating the pathophysiology of PD, the processes of clinical diagnosis and therapeutic intervention continue to be inadequate, encountering considerable obstacles such as the heterogeneity of the disease (Sveinbjornsdottir, 2016). Insomnia is one of several common non-motor symptoms of PD, affecting a majority of patients and often co-existing with circadian dysregulation and excessive daytime sleepiness (Henderson, 2022). Insomnia is also considered a common comorbidity among PD patients, which is caused by degeneration of the neural structures that modulate sleep (Iranzo et al., 2024). At the molecular level, classical PD phenotypes, such as neuroinflammation, mitochondrial stress, and impaired proteostasis, are central to PD pathogenesis and can be modulated by sleep and circadian homeostasis (Iranzo et al., 2024). Hence, deciphering the cellular and molecular level of insomnia in PD may facilitate the development of novel therapeutic strategies aimed at improving insomnia and increasing the quality of life for PD patients.

**Figure 1:**
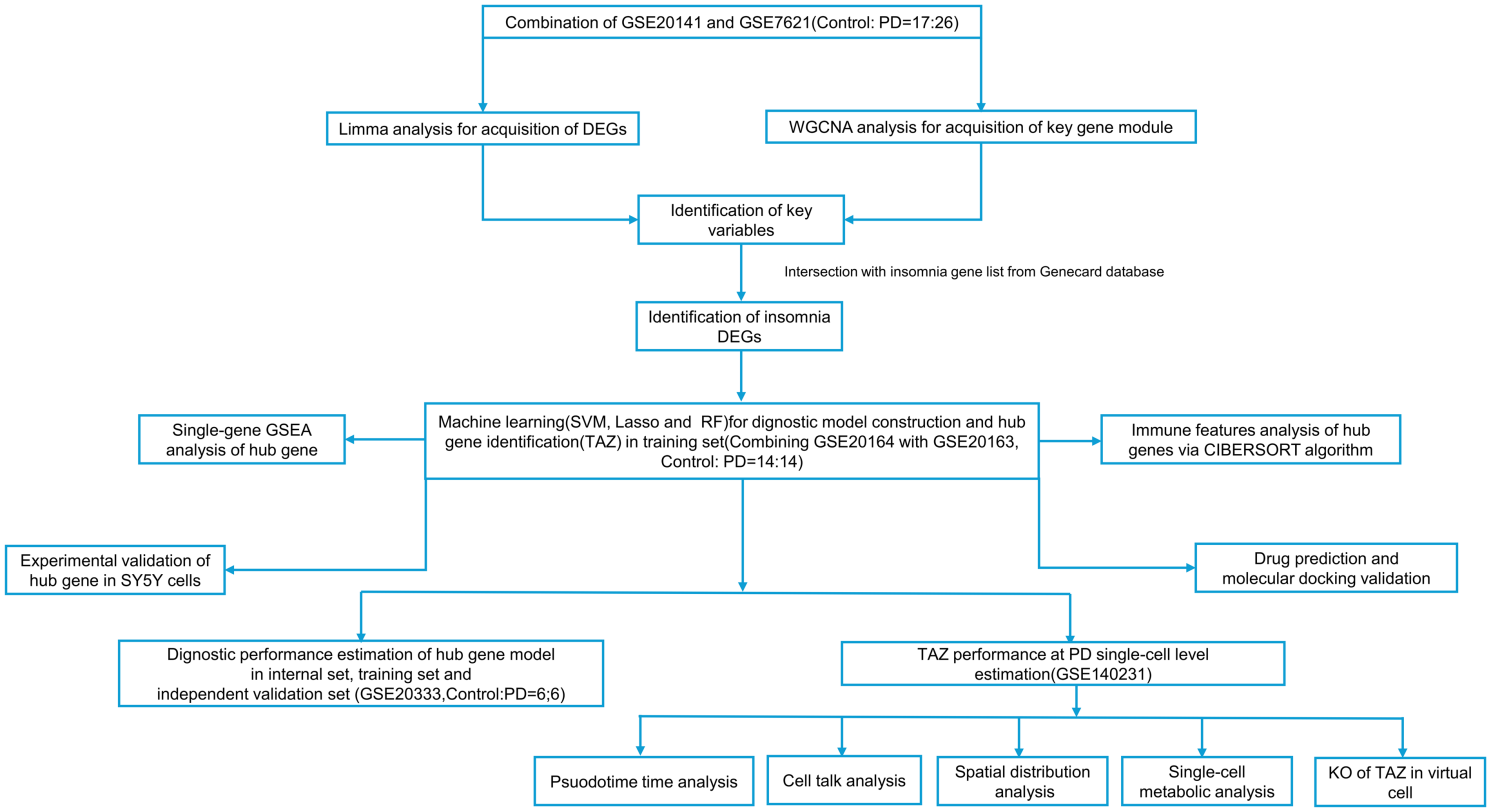
Workflow of this study.

In this study, we used artificial intelligence (AI) with integrative bioinformatics pipelines and multi-omics to discover molecular mechanisms and therapeutic strategies for the treatment of PD. First, the internal public bulk-seq dataset (integrated GSE20141 and GSE7621 bulk profile, including 17 Control and 26 PD SN samples) was analyzed for the identification of co-expression differentially expressed gene (DEG) using limma and WGCNA analysis. The insomnia-related gene list was intersected with co-expression DEGs to render insomnia-related DEGs (IDEGs). Based on IDEGs, the trained public bulk profile (integrated GSE20163 and GSE20164, including 15 control and 14 PD SN samples) was utilized to identify insomnia-associated hub genes using three machine learning algorithms (random forest [RF], least absolute shrinkage and selection operator [LASSO], and support vector machine [SVM]). The results indicated that TAZ can be considered as a hub gene involved in PD pathogenesis. An independent validation set (GSE20333, including six control and six PD SN samples) along with internal and training sets were utilized for TAZ PD diagnostic performance evaluation using ROC, PR, DCA, nomogram, and calibration analyses. The results indicated that TAZ could be considered a favorable diagnostic biomarker for PD onset. Furthermore, single-cell analysis revealed that *TAZ* is mainly distributed in neurons and associated with neuronal differentiation and PD pathogenesis. Furthermore, an AI-driven therapeutic screening framework identified BRD-K98481123 as a potential therapeutic agent for PD treatment by targeting TAZ based on the PD training bulk profile. Finally, *in vitro* studies demonstrated that *TAZ* was up-regulated. Our study is the first to report the diagnostic role of insomnia-related TAZ in PD and offers novel clinical therapeutic strategies. We describe the workflow of this study in Figure 1.

## 2 Materials and methods

### 2.1 Source of data

We first downloaded five SN microarray datasets of PD patients and corresponding clinical information from the Gene Expression Omnibus (GEO) database using the GEOquery package of R software, including GSE20141, GSE20163, GSE20164, and GSE20333 (Davis and Meltzer, 2007). We integrated GSE20141 and GSE7621 (based on GPL570) using the sva package of R software for removing a batch effect, and considered this integration as an internal set (Leek et al., 2012). Next, GSE20163 and GSE20164 (based on GPL96) were considered as the training set and subjected to integration following the same rules. Furthermore, GSE20333 (based on GPL201) was considered as an independent validation set. All these datasets were normalized and standardized using the limma package of R (Ritchie et al., 2015). The insomnia-related gene list was acquired from the Genecard database with a threshold > 1(Xu et al., 2023).

### 2.2 Identification of DEGs and WGCNA analysis

Differential expression analysis was performed on the internal set using the limma package or R software (Ritchie et al., 2015). DEGs were identified using thresholds of | log_2_FC | > 1 and *p* < 0.05 and visualized with a volcano map and a heatmap using ggplot2 and complexheatmap packages (Gustavsson et al., 2022; Gu, 2022). Furthermore, we utilized the WGCNA package in R software to investigate the association between genes and phenotypes by constructing a gene co-expression network in the internal set (Langfelder and Horvath, 2008). Initially, we excluded 50% of the genes with the lowest median absolute deviation (MAD) (Langfelder and Horvath, 2008). Following this, we calculated Pearson’s correlation matrices for all possible gene pair comparisons and constructed a weighted adjacency matrix by applying the average linkage method in conjunction with a weighted correlation coefficient (Langfelder and Horvath, 2008). The “soft” thresholding power (*β*) was subsequently utilized to ascertain the adjacency, which was then converted into a topological overlap matrix (TOM) (Langfelder and Horvath, 2008). To group genes with similar expression patterns into modules, we performed average linkage hierarchical clustering based on the dissimilarity metric derived from the TOM, ensuring a minimum group size of 50 genes (Langfelder and Horvath, 2008). Ultimately, we evaluated the dissimilarity of module eigengenes, established a cutoff for the module dendrogram, and merged several modules. The WGCNA was used to identify significant modules associated with PD, resulting in the development of a visualized eigengene network. In addition, the insomnia-related gene list was intersected with DEGs acquired from limma and the co-expression module acquired from WGCNA for the identification of IDEGs, which was visualized by a Venn plot generated by R software (Jia et al., 2021). Furthermore, the KEGG and GO functional enrichment analyses of IDEGs was performed using the clusterProfiler in R with a threshold FDR < 0.05 in accordance with the hallmark gene set downloaded from the MSIGDB database (Yu et al., 2012; Liberzon et al., 2015).

### 2.3 Machine learning algorithms and diagnostic model construction

LASSO logistic regression analysis represents a sophisticated data mining technique that utilizes an L1 penalty (lambda) to effectively minimize the coefficients of less critical variables to zero (Kang et al., 2021). This approach enables the identification of significant variables, facilitating the development of an optimal classification model. The SVM-RFE approach is a supervised machine learning methodology used to ascertain the most critical core genes by systematically eliminating feature vectors produced by the Support Vector Machine (Guan et al., 2024). Random forest (RF) analysis is a decision tree-based machine learning method that focuses on evaluating the significance of variables by scoring the importance of each variable (Wallace et al., 2023). In combination with these three machine learning algorithms with a training set, we acquired the insomnia hub variable involved in PD pathogenesis. Next, the hub variable molecular function was assessed in the training set using the single-gene GSEA analysis in accordance with the hallmark gene set downloaded from the MSIGDB database via the clusterProfiler package of R (Yu et al., 2012). Furthermore, the immune feature of the hub variable was estimated by the CIBERSORT algorithm of R (Chen et al., 2018). Next, the expression and diagnostic value of the hub variable were also cross-validated in internal, training, and independent sets. Diagnostic performance of the hub variable was calculated by ROC, PR, DCA, nomogram, and calibration using pROC, rms, and rmda packages of R software (Robin et al., 2011; Lin et al., 2024; Liu et al., 2024).

### 2.4 Single-cell transcriptomic analysis

We retrieved the single-cell transcriptomic dataset associated with SN in PD patients, specifically GSE140231, from the GEO database. The analysis of the single-cell RNA sequencing (scRNA-seq) data involved several essential steps, including quality control (QC), dimensionality reduction, and identification of markers, all of which were performed using the Seurat R package (Butler et al., 2018). A strict quality control process was implemented for each cell, adhering to predefined criteria that stipulated gene counts should range from 200 to 6,000, the count of unique molecular identifiers (UMIs) should surpass 1,000, and the percentage of mitochondrial genes should remain below 10% (Butler et al., 2018). Upon completion of these QC procedures, the data were normalized, enabling the identification of 2000 genes that demonstrated significant variability for further analysis (Butler et al., 2018). Following normalization, dimensionality reduction methods, particularly t-SNE and UMAP, were applied. Cell type annotations were conducted utilizing the scMayoMap algorithm implemented in the R software (Yang et al., 2023). We evaluated the expression levels of the target genes across the various annotated cell populations. Intercellular communication networks were inferred through the use of the CellTalker package in R (Barut et al., 2022). Furthermore, we investigated energy metabolic pathways at the single-cell level among the annotated cell populations by using the scMetabolism package in R (Argüello et al., 2020). Importantly, a pseudo-time analysis of the expression of targeted genes within specific cell types was performed in both temporal and spatial contexts using the monocle2 package in R (Fang et al., 2022). ScTenifoldKnk was performed for the identification of Knockout (KO) of the hub gene in the targeted cell (Osorio et al., 2022).

### 2.5 AI-driven drug prediction and molecular docking

The DrugRefLector framework, which uses active learning to utilize transcriptomic data, was utilized to identify modulators associated with disease phenotypes (DeMeo et al., 2025). Utilizing the integrated GSE20164 and GSE20163 bulk profiles, we implemented DrugRefLector to discover optimal therapeutic agents aimed at alleviating PD (DeMeo et al., 2025). To evaluate the binding affinity of the optimal therapeutic agents to the hub gene, we conducted molecular docking studies (Wang et al., 2022). This molecular docking was crucial for examining the interactions between the selected drugs and their corresponding proteins (Wang et al., 2022). The Protein Data Bank (PDB) files for the target proteins (PDB ID: 5 N75) were obtained from the RCSB PDB repository, while ligand SDF files (Pubchem ID: 44620789) were sourced from the PubChem database (Berman et al., 2000; Kim, 2016). Following this, we executed molecular docking to quantify the binding affinities between the target proteins and the compounds. Initially, PyMOL software (Version 2.6.0) was used to remove water molecules and ligands, retaining the protein backbone (Ji et al., 2023). Subsequently, the AutoDock Vina Tool (Version 4.2.6) was utilized to identify potential binding sites on the protein surface and to perform flexible molecular docking (Ji et al., 2023). This process entailed calculating docking scores and binding affinities (expressed as Vina scores in kcal/mol) for each identified binding site (Ji et al., 2023). We ranked the top five binding sites based on the calculated binding energy, ultimately selecting the site with the lowest energy for visualization in PyMOL. This visualization enabled us to identify the locations of hydrogen bonds associated with ligand binding in the resulting images (Ji et al., 2023). The outcomes were subsequently illustrated in PyMOL to demonstrate binding modes and hydrogen bonding interactions (Ji et al., 2023).

### 2.6 Cell lines and culture conditions

Authenticated human dopaminergic (DA) neuron SH-SY5Y cells were obtained from the Shanghai Institute of Cell Biology (Shanghai, China). These cells were cultured in Dulbecco’s modified Eagle medium (DMEM), supplemented with 10% fetal bovine serum (FBS) and 1% penicillin–streptomycin. The cultures were maintained at 37 °C in a humidified incubator containing 5% CO₂ (Quan et al., 2024). The medium was refreshed every 2–3 days, and the cells were passaged when they reached approximately 80% confluence (Quan et al., 2024). To simulate neuronal injury, SY5Y cells were exposed to ultrapure MPP + (200 mM; #D048, Sigma-Aldrich, St. Louis, MO, USA) for a duration of 24 h at 37 °C. MPP^+^ SY5Y cells were cultured for simulated PD, and SY5Y cells were cultured as a normal control (Quan et al., 2024).

### 2.7 Quantitative real-time PCR (qRT-PCR)

Total RNA was isolated using the TRIzol reagent (TaKaRa, Beijing, China), and the subsequent analysis of its concentration, purity, and integrity was performed utilizing a NanoDrop spectrophotometer (Thermo Scientific, Waltham, MA, USA) (Zhou et al., 2024). Reverse transcription was executed with 1 μg of total RNA using HiScript II Q RT SuperMix for qPCR (+gDNA wiper) alongside a gDNA eraser (Vazyme, Shanghai, China) (Zhou et al., 2024). The concentration, purity, and integrity of the resultant cDNA were also assessed using a NanoDrop spectrophotometer (Thermo Scientific, Waltham, MA, USA) (Zhou et al., 2024). Quantitative reverse transcription PCR (qRT-PCR) was carried out with SYBR Green MasterMix (11203ES50, YEASEN, Shanghai, China) and analyzed through StepOne Software v.2.3 (Applied Biosystems, Carlsbad, CA, USA), incorporating 40 amplification cycles (three biological replicates) (Zhou et al., 2024). Data analysis was performed using the ∆∆Ct (cycle threshold) method, with normalization to the expression levels of the reference gene, GAPDH. The primer sequences for the target gene were as follows:

*TAZ*:

Forward: 5′-GACCCCAGACATGAGATCCA-3′.

Reverse: 5′-CCTGCGTTTTCTCCTGTATCC-3′.

GAPDH:

Forward, 5′-GAGAAGGCTGGGGCTCATTT-3′.

Reverse, 5′-ATGACGAACATGGGGGCATC-3′.

### 2.8 Statistical analysis

All statistical analyses were performed in R software (Version 4.2.2) and GraphPad Prism (version 9.0). Differences between the two groups were assessed using Student’s *t*-test or the Wilcoxon rank-sum test, depending on data distribution. Comparisons among multiple groups were conducted using the Student t-test and one-way ANOVA, followed by Tukey’s *post-hoc* test. Correlations between gene expression and immune cell infiltration were evaluated using Spearman’s correlation analysis. A two-tailed *p*-value of < 0.05 was considered statistically significant.

## 3 Results

### 3.1 Identification of proposed variables in PD patients using limma and WGCNA analysis

**Figure 2:**
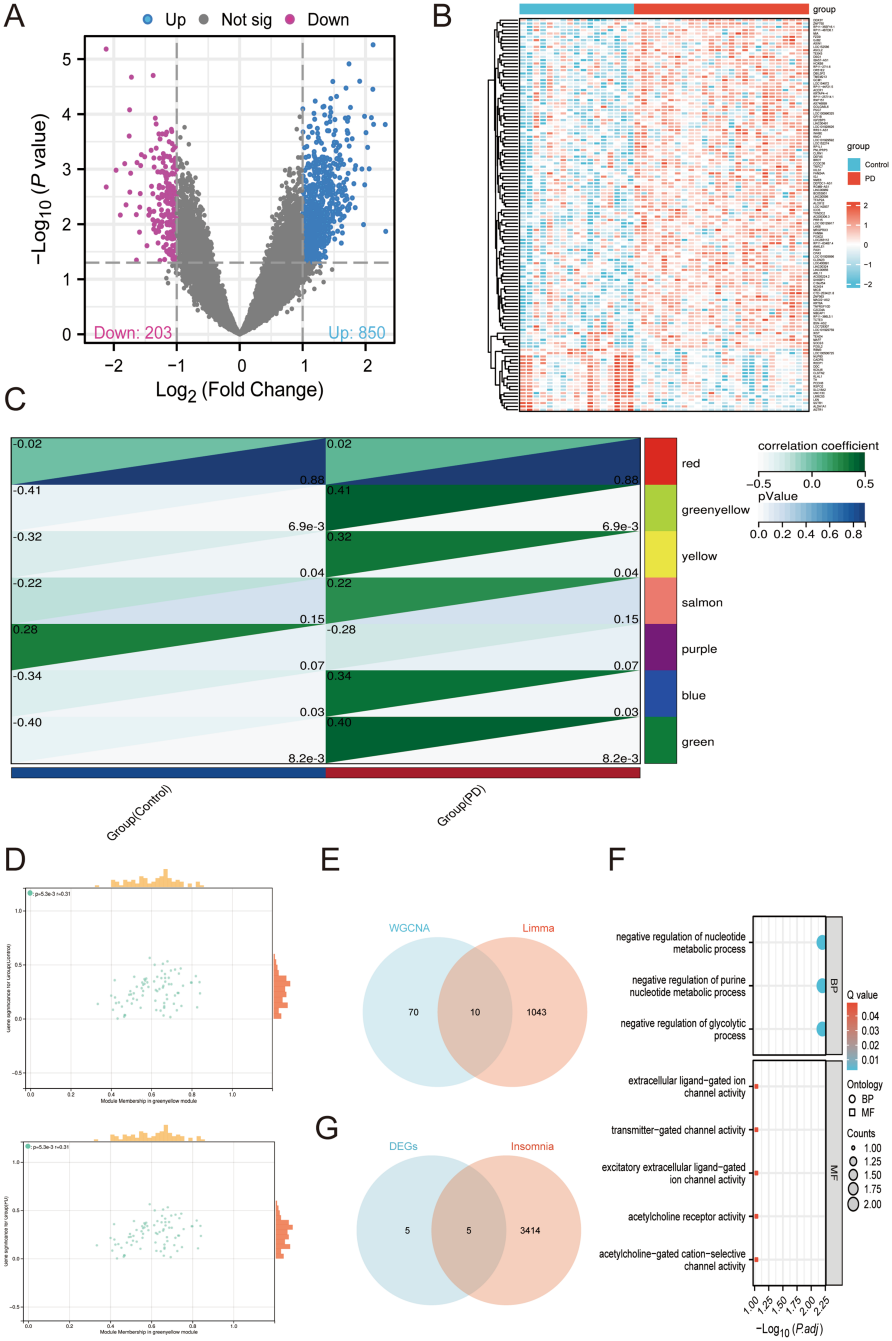
Identification of insomnia-associated DEGs in PD patients. **(A)** Volcano plot shows DEGs between PD patients and healthy controls in the internal set. **(B)** Heatmap illustrates the expression patterns of distinct DEGs in the internal set. **(C)** Module trait relationship heatmap generated by WGCNA in the internal set. **(D)** Scatterplots of representative modules associated with PD and control in the internal set. **(E)** Venn diagram depicts the overlap between DEGs identified by limma and the co-expression gene module derived from WGCNA. **(F)** KEGG and GO enrichment analysis of insomnia-related DEGs. **(G)** Venn diagram shows the intersection between targeted DEGs and the insomnia gene list.

To identify candidate genes associated with Parkinson’s disease (PD), we first performed quality control and normalization of the internal set. Principal component analysis (PCA) revealed a clear separation trend between PD patients and healthy controls (Supplementary Figure S1A). Boxplots after normalization confirmed consistent expression distributions across samples (Supplementary Figure S1B). A total of 1,053 DEGs were identified, including 850 upregulated and 203 downregulated genes (Figure 2A). Heatmap analysis further illustrated distinct expression profiles between PD patients and controls (Figure 2B). Using weighted gene co-expression network analysis (WGCNA), a soft-threshold power of *β* = 4 was selected to achieve a scale-free topology (Supplementary Figure S1D). Hierarchical clustering of genes allowed the construction of multiple co-expression modules (Supplementary Figure S1C), and their interrelationships were further visualized through a module clustering heatmap (Supplementary Figure S1E). By correlating co-expression modules, we found that the green-yellow modules were most strongly associated with PD (Figures 2C,D). To enhance robustness, we intersected the DEGs obtained from limma and PD-associated WGCNA modules with the insomnia-related gene list, and identified 5 overlapping genes (Figures 2E,G). Functional enrichment analysis revealed that these intersected genes were significantly enriched in biological processes such as nucleotide metabolic processes, purine nucleotide metabolism, and glycolytic pathways, while molecular function analysis highlighted their roles in ligand-gated ion channel activity and cholinergic receptor activity (Figure 2F).

### 3.2 Insomnia-related diagnostic signature and hub gene identification for PD patients using machine learning

**Figure 3:**
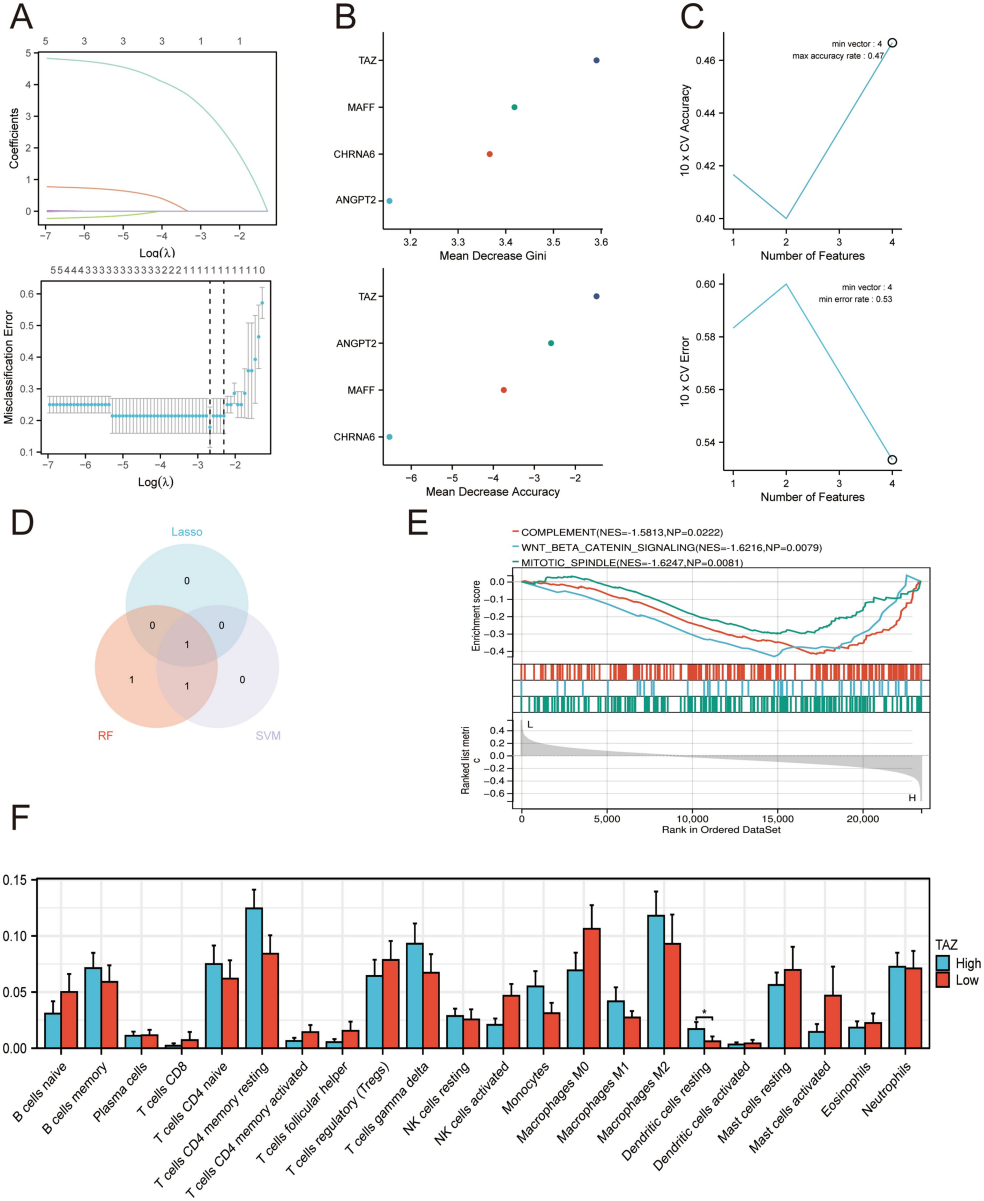
Identification of an insomnia-related hub gene for PD using machine learning approaches. (A) LASSO regression analysis for feature selection among candidate DEGs. **(B)** RF analysis ranks the importance of candidate genes. **(C)** SVM-RFE curves show cross-validation accuracy and error rate across different feature subsets. **(D)** Venn diagram illustrates the intersection of candidate genes identified by LASSO, RF, and SVM. **(E)** Single-gene GSEA analysis of TAZ. **(F)** CIBERSORT-based immune cell infiltration analysis of TAZ.

To construct an insomnia-related hub gene linked with PD, we applied LASSO, RF, and SVM-RFE for hub variable identification (Figures 3A–C). Integrative analysis of the 3 methods revealed 1 overlapping hub gene, TAZ (Figure 3D). To explore the molecular role of TAZ, we performed single-gene GSEA. The results demonstrated that TAZ was significantly associated with signaling pathways, including complement and immune response, WNT/β-catenin signaling, and mitotic spindle regulation (Figure 3E). Furthermore, CIBERSORT immune cell infiltration analysis indicated that TAZ expression was negatively correlated with the dendritic cell resting proportion (Figure 3F).

### 3.3 Cross-validation of insomnia-related diagnostic model performance in PD patients

**Figure 4:**
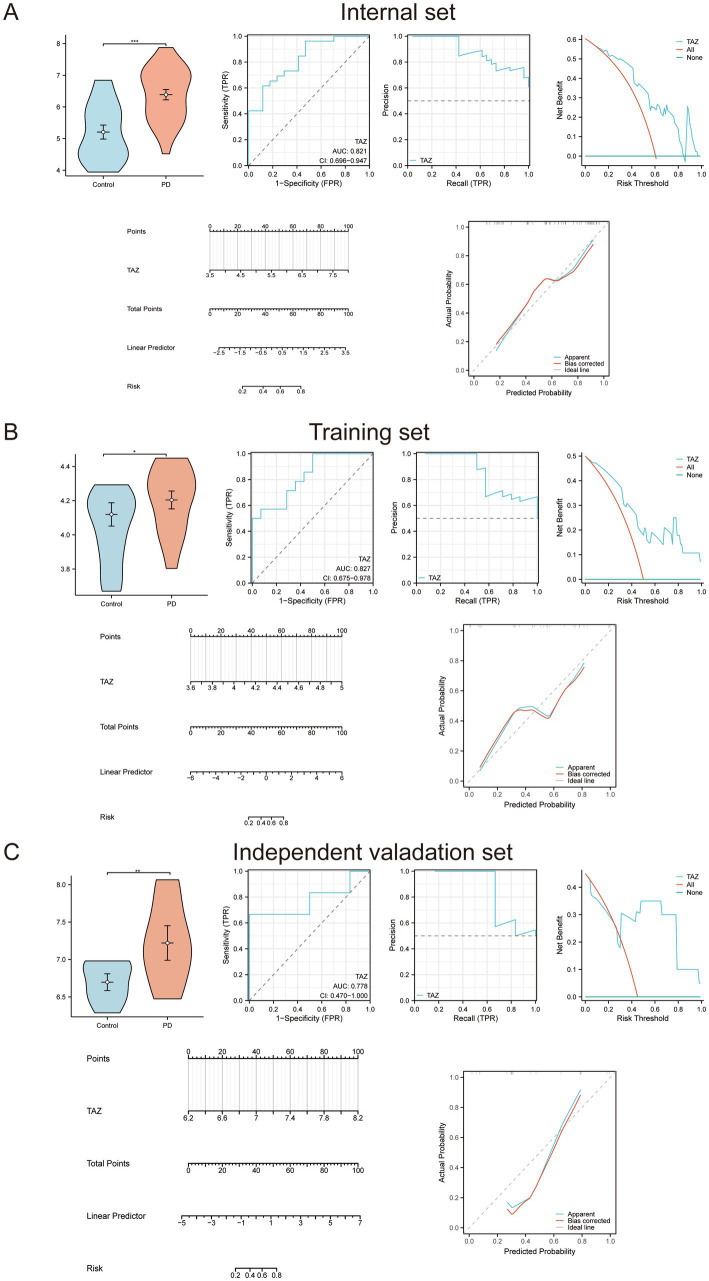
Cross-validation of the diagnostic performance of TAZ in PD. **(A)** Expression and diagnostic value evaluation of TAZ in the internal set. **(B)** Expression and diagnostic value evaluation of TAZ in the training set. **(C)** Expression and diagnostic value evaluation of the independent validation set.

We next validated the diagnostic performance of TAZ for PD. Expression analysis revealed significantly higher TAZ expression levels in PD patients compared to healthy controls across all datasets (Figures 4A–C). To assess diagnostic efficacy and accuracy of TAZ, ROC, PR DCA, nomogram, and calibration were utilized across internal, training, and independent validation sets. Collectively, the findings illustrate that TAZ serves as a robust biomarker with favorable diagnostic performance for PD (Figures 4A–C).

### 3.4 Landscape of *TAZ* at the single-cell level in PD patients

**Figure 5:**
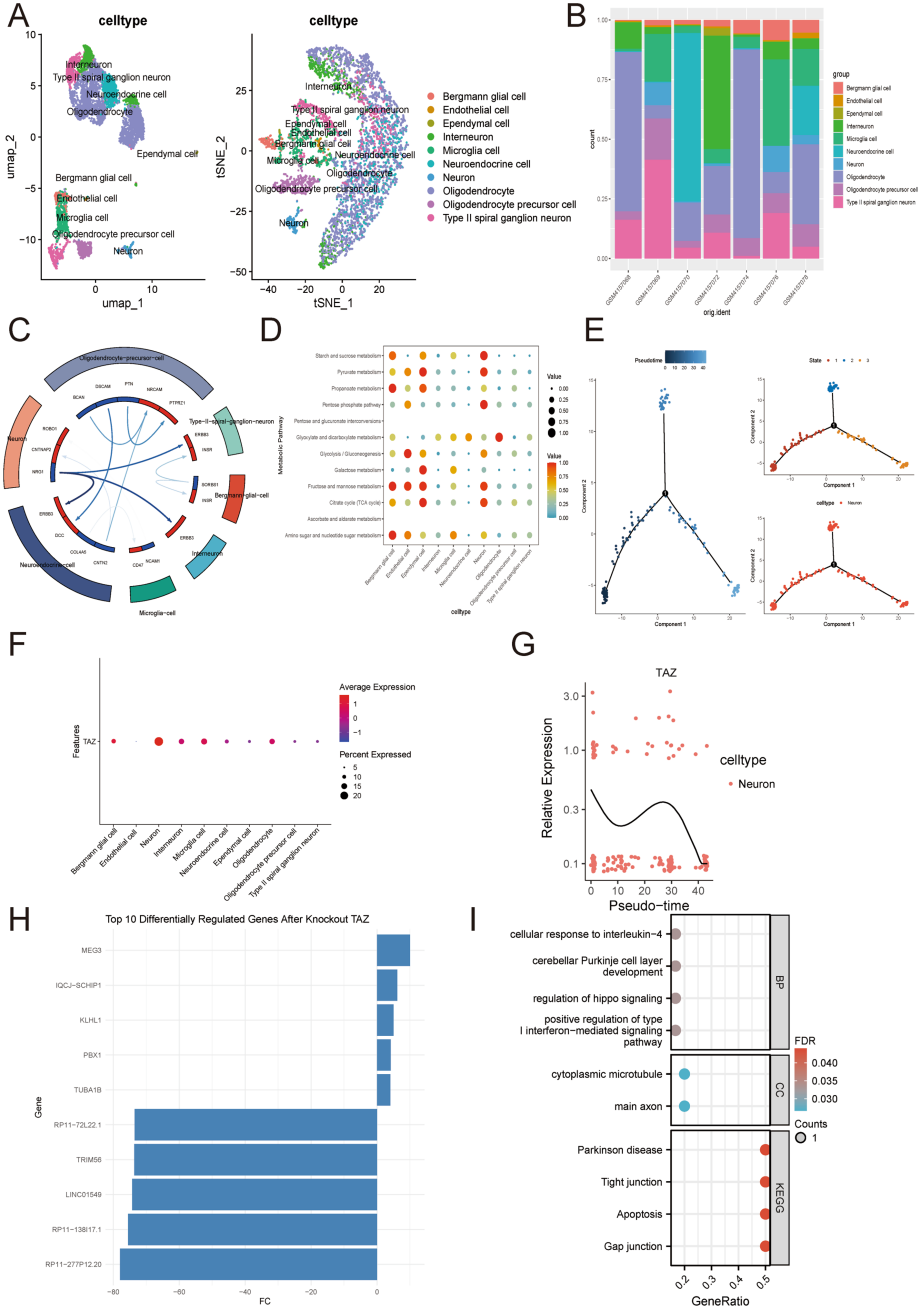
Global single-cell analysis of TAZ in PD patients. **(A,B)** UMAP and t-SNE plots display 10 annotated cell types and corresponding cell proportions across 7 samples. **(C)** Cell–cell communication network. **(D)** Metabolic heterogeneity among these 10 cell types. **(E)** Differentiation patterns of neurons. **(F)** Distribution of TAZ among these 10 cell types. **(G)** Pseudotime trajectory analysis of TAZ in neurons. **(H,I)** AI virtual KO of TAZ in neurons.

To further elucidate the heterogeneity of SN and trace the temporal and spatial mechanisms of TAZ for PD patients, we performed single-cell RNA sequencing analysis in GSE140231 (including seven SN samples from PD patients). Rigorous QC confirmed stable sequencing depth, gene counts, and mitochondrial content across samples (Supplementary Figures S2A,B). Heatmap visualization of marker gene expression enabled accurate cell-type annotation, ultimately identifying 19 main cell clusters (Supplementary Figures S2D,E). For annotation, both UMAP and t-SNE plots illustrated distinct 10 cell types (Figure 5A). Significantly, oligodendrocytes and interneurons shared the largest proportion, indicating the persistent neuroinflammatory and neurodegenerative signaling in PD patients (Ma et al., 2025; Panicker et al., 2022) (Figure 5B). Next, cell chat manners among these 10 cell types and the corresponding ligand-receptor were analyzed (Figure 5C). In addition, we also discovered metabolic heterogeneity between these 10 cell types (Figure 5D). TAZ is mainly distributed in neurons and is involved in the neuronal cell cycle (Figure 5F). Pseudotime trajectory analysis suggested that TAZ dynamically regulates neuronal differentiation, with expression peaking during early-to-mid developmental stages and declining in terminally differentiated neurons (Figures 5E,G). In virtual neurons, we performed KO of TAZ and illustrated the Top10 DEGs after TAZ (Figure 5H). Indeed, these 10 DEGs were mainly involved in various pathways and functions closely related to PD pathogenesis, indicating that *TAZ* a crucial role in PD progression (Figure 5I).

### 3.5 *In vitro* examination of TAZ expression in SY5Y cells and identification of potential therapeutic agents

**Figure 6:**
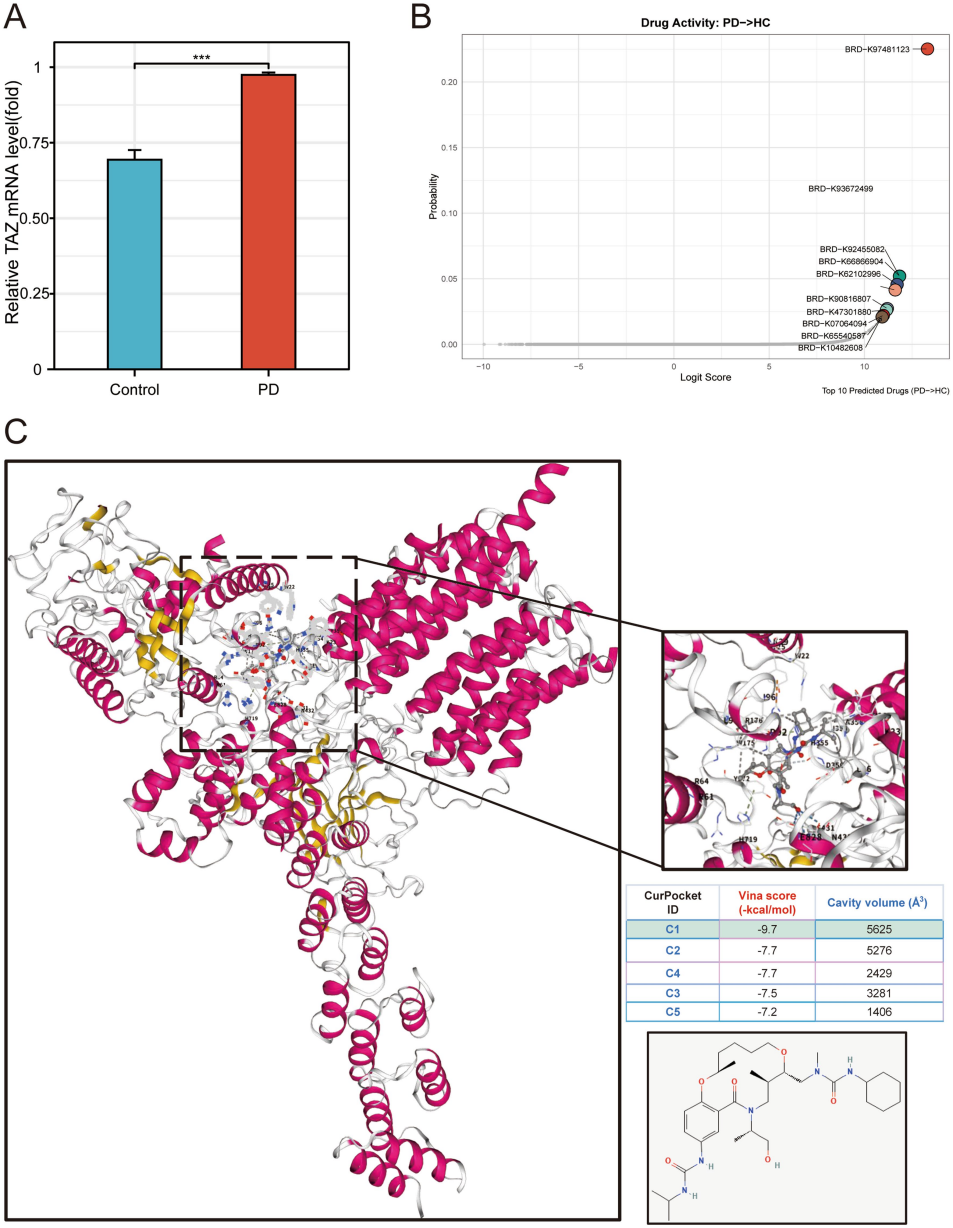
Experimental validation of TAZ expression and identification of potential therapeutic agents for PD treatment. **(A)** q-RT-PCR analysis of TAZ expression levels between MPP^+^ SY5Y cells and SY5Y cells. **(B)** Candidate drug screening results from DrugRefLector prediction. **(C)** Molecular docking between BRD-K98481123 and TAZ.

To experimentally validate the relevance of TAZ in Parkinson’s disease (PD), we measured its expression in MPP^+^ SY5Y cells and SY5Y cells. The results showed that TAZ mRNA expression was significantly upregulated in PD cells compared to controls (Figure 6A). To further investigate potential therapeutic agents targeting TAZ, we utilized an AI-driven therapeutic screening framework (DrugRefLector) in an integrated GSE20164 and GSE20163 bulk profile (training set)(Figure 6B). Results have shown that 10 agents can potentially reverse PD, and BRD-K98481123 was the optimal one (Figure 6B). To assess whether TAZ can be considered as a target for BRD-K98481123, we performed molecular docking validation (Figure 6C). Results indicated that BRD-K98481123 can bind to TAZ C1 cavity pocket with favorable binding affinity (−9.7 kcal/mol). These results indicated that TAZ plays a pathogenic role in PD progression and BRD-K98481123 can be considered as a potential therapeutic agent for PD treatment by targeting TAZ.

## 4 Discussion and conclusion

In this study, we systematically integrated bulk and single-cell transcriptomics with an artificial intelligence (AI) framework for insomnia-related predictive and therapeutic model construction for PD patients. Our findings identified that TAZ can be considered an upregulated diagnostic and druggable target for PD patients. Single-cell analysis revealed that TAZ was mainly distributed in neurons and involved in biological functions and pathways related to the PD pathogenesis.

TAZ (WWTR1), a Hippo pathway effector, was mainly located in the cytosol and the nuclear body. For neuro-regulation, TAZ exhibits distinct roles in modulating the differentiation of astrocytes (Chen et al., 2024). Specifically, TAZ plays a crucial role in overseeing the process of differentiation and maturation as these progenitors transition into fully developed astrocytes (Chen et al., 2024). Furthermore, overexpression of TAZ also contributes to the progression of Glioblastoma using activation of cariogenic signals (Pontes and Mendes, 2023). Furthermore, reports have been verified that modulation of Hippo signals can alleviate the motor and non-motor symptoms and restore cognition for PD patients (Choi et al., 2024). Indeed, insomnia, a major non-motor complication for PD patients, significantly affects PD patients’ quality of life (Henderson, 2022). Previous investigations indicated that imbibition of the hippo signaling modulator can reduce neuroinflammation and improve insomnia in an animal model (Choi et al., 2024). However, the definite role of TAZ in PD pathogenesis and the corresponding mechanisms of TAZ in regulating insomnia have not yet been elucidated.

Overall, in this study, our integrative approach highlights the mechanistic links between insomnia and PD progression at the molecular level using AI pipelines and multi-omics. We also elucidated insomnia and Hippo-related TAZ as an up-regulated diagnostic biomarker and potential therapeutic target, with corresponding molecular and immune features involved in PD progression. Indeed, BRD-K98481123 can be considered as a potential therapeutic agent targeting TAZ for the treatment of PD. However, there are several limitations to our study. First, the TAZ mechanisms of TAZ in PD pathogenesis acquired *in silico* should be verified in a pre-clinical study. For example, the prediction of scTenifoldKnk may favor regulatory rather than structural genes, as the latter tend to have a smaller degree in the network (Osorio et al., 2022). Hence, *in silico* KO of TAZ accuracy should be validated in real-world experiments to enhance the robustness of the results. Second, the therapeutic efficacy of BRD-K98481123 targeting PD should be assessed by pre-clinical and clinical studies. Furthermore, the diagnostic performance of TAZ targeting PD should be verified in multi-center studies. Future research should be focused on addressing the mechanisms of TAZ involved in PD pathogenesis and its therapeutic potential.

## Data Availability

The datasets presented in this study can be found in online repositories. The names of the repository/repositories and accession number(s) can be found at: https://www.ncbi.nlm.nih.gov/geo/, GSE20141; https://www.ncbi.nlm.nih.gov/geo/, GSE7621; https://www.ncbi.nlm.nih.gov/geo/, GSE20164; https://www.ncbi.nlm.nih.gov/geo/, GSE20163; https://www.ncbi.nlm.nih.gov/geo/, GSE20333; https://www.ncbi.nlm.nih.gov/geo/, GSE140231.
