# Supplementary material for: Artificial intelligence and multi-omics nominate TAZ as an insomnia-related diagnostic and druggable target for Parkinson’s disease patients
Source: Front Aging Neurosci. 2026 Feb 4;18:1727472. doi: 10.3389/fnagi.2026.1727472 (PMC12913377; doi:10.3389/fnagi.2026.1727472)
Supplement: Supplementary file 1 [file Supplementary_file_1.docx]

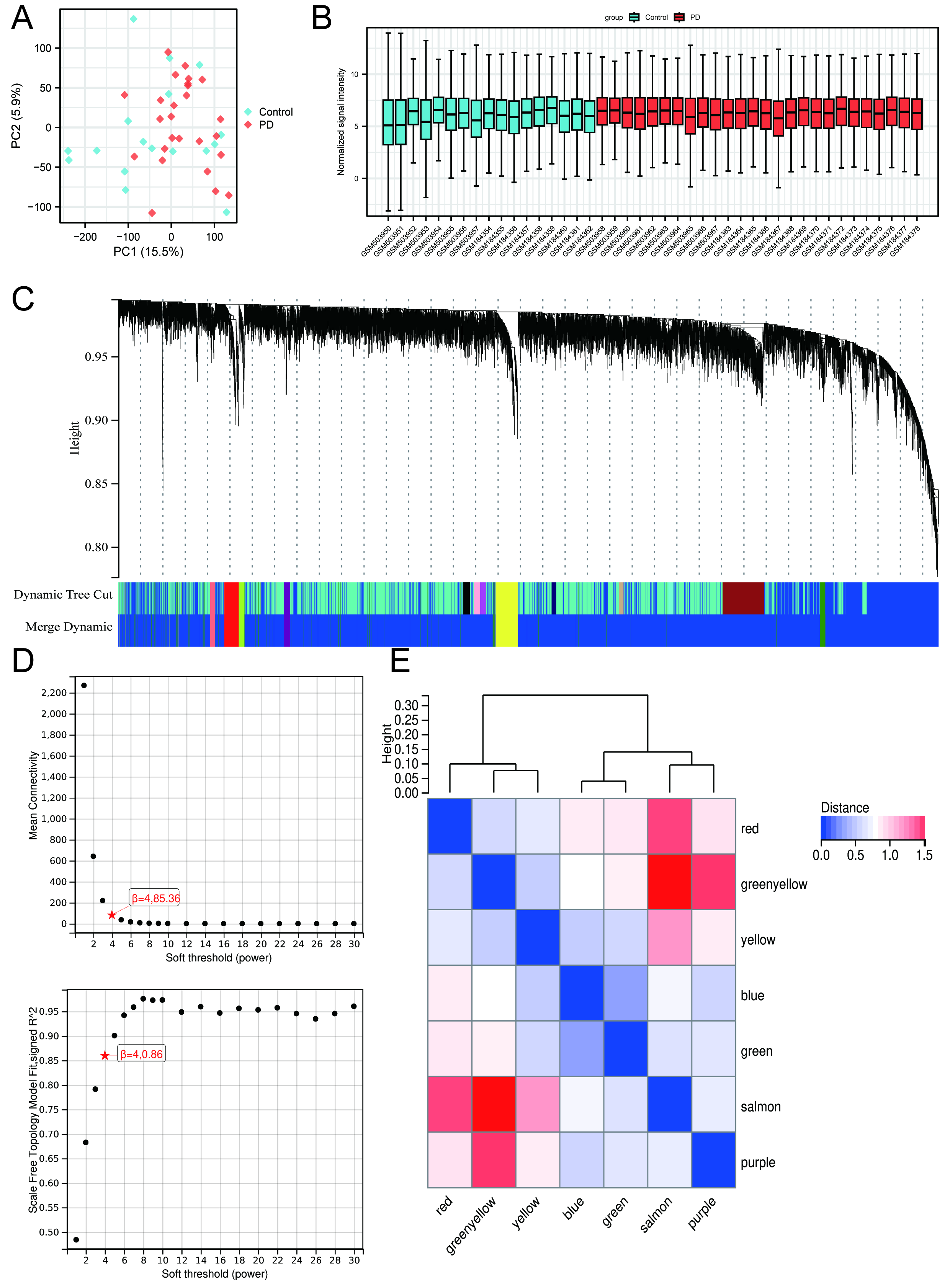


**Figure S1. Construction of co-expression modules using WGCNA**. **(A)** PCA showing partial separation between PD and control samples. **(B)** Boxplots of normalized gene expression data across samples **(C)** Dendrogram of genes clustered by WGCNA, with different colors representing distinct gene modules. **(D)** Analysis of network topology to determine the soft-thresholding power. **(E)** Heatmap of eigengene adjacency illustrating the relationships among different gene modules.


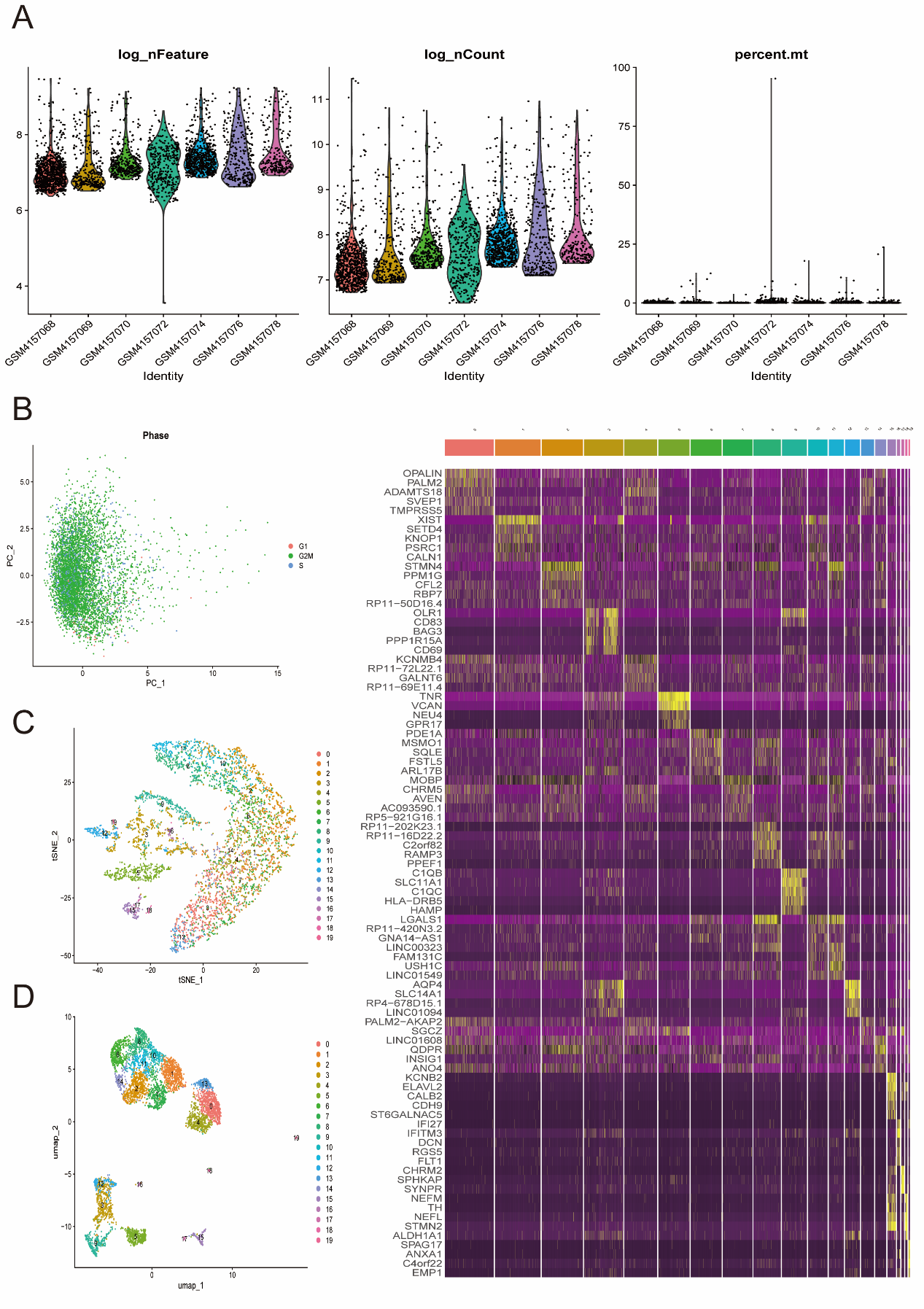


**Figure S2. Quality control and clustering of single-cell transcriptomic data. (A)** QC metrics of single-cell data across samples. **(B)** PCA analysis of cells based on cell cycle phase (G1, S, G2M). **(C-D)** Dimensionality reduction using t-SNE and UMAP, revealing distinct cell clusters. **(E)** Heatmap illustration of marker in different clusters.
